# Gα_i_2 Induces Cell Migration in PC3 Prostate Cancer Cells in the Absence of Rac1 Activation

**DOI:** 10.3390/ijms26062663

**Published:** 2025-03-15

**Authors:** Rarnice Johnson, Silvia Caggia, Shafiq A. Khan

**Affiliations:** 1 Center for Cancer Research and Therapeutic Development and Department of Biological Sciences, Clark Atlanta University, 223 James P. Brawley Dr, Atlanta, GA 30314, USA; rarnice.johnson@students.cau.edu (R.J.); sxc2803@miami.edu (S.C.); 2 Department of Microbiology and Immunology, Miller School of Medicine, University of Miami, Coral Gables, FL 33101, USA

**Keywords:** prostate cancer, cell migration, Rac1, lamellipodia, Gα_i_2

## Abstract

Metastatic prostate cancer occurs when the tumor spreads from the prostate gland to other parts of the body. Previous studies have shown that Gα_i_2, a subunit of the heterotrimeric G protein complex, plays a critical role in inducing cell migration and invasion in prostate cancer cells in response to diverse stimuli. Rac1 is a small rho-GTPase, which is activated by the phosphoinositide 3-kinase (PI3K)/AKT pathway and plays an essential role during cell migration. Previous studies have shown that the knockdown of Gα_i_2 attenuates cell migration without causing any reduction in basal Rac1 activity in both PC3 and DU145 cells, and has only marginal effects on the epidermal growth facotor (EGF)-induced increase in Rac1 activity. Therefore, Gα_i_2 may be involved in the regulation of cell motility and invasion independently or downstream of Rac1 activation. In this study, we investigated the possible mechanism of Gα_i_2 at the level of the Rac1-dependent activation of Wiskott-Aldrich Syndrome Protein)-family verprolin homologous protein2 (Wave2) and actin related protein 2/3 (Arp 2/3) proteins, downstream effectors of activated Rac1. PC3 cells with a stable overexpression of constitutively active Rac1 were transfected with control siRNA or Gα_i_2 siRNA to knockdown endogenous Gα_i_2 expression. Western blot analysis showed that the Rac1-dependent activation of Wave2 was impaired in the absence of Gα_i_2. The overexpression of constitutively active Gα_i_2 (Gα_i_2-Q205L) in PC3 cells significantly increased cell migration compared to cells transfected with control plasmids. In the parallel experiments, a specific Gα_i_2 inhibitor blocked G_i_α2-Q205L-induced cell migration in PC3 cells. Furthermore, the Rac1 inhibitor did not block increased cell migration in PC3 cells overexpressing constitutively active Gα_i_2. We conclude that activated Gα_i_2 plays a crucial role in cell migration in prostate cancer cells independent of Rac1 activation.

## 1. Introduction

Prostate cancer is the most common cancer in American men. According to the American Cancer Society, there will be nearly 299,010 new cases of prostate cancer and about 35,250 deaths due to prostate cancer in 2024. Early-stage prostate cancer is localized in the prostate gland and is treatable through surgery and radiation therapy [1,2]. However, metastatic prostate cancer, which has spread to distant tissues including bones, causes significant problems for treatment [1,2].

Metastasis is a multistep process during which tumor cells escape from the primary sites, spread through lymphatic and/or blood circulations, and ultimately disseminate to distant sites [3,4]. Among these complex processes, one of the most critical steps is tumor cell motility or cell migration, which is regulated by numerous intracellular proteins [5]. The RHO family of GTPases, including RhoA, Rac1, and Cdc42, plays a pivotal role in the regulation of directed cell migration [5]. During cell migration, the formation of actin-driven protrusion at the leading edge, called lamellipodia, is regulated by Rac1 activation, whereas focal adhesion and the formation of stress fibers at the cell body and the rear of the cells are regulated by RhoA activation; filopodia formation, which establishes cell polarity, is regulated by Cdc42 [5].

Cell migration is regulated by several growth factors and cytokine through the activation of several membrane receptors, including receptor tyrosine kinases and G-protein coupled receptors (GPCRs) [6]. The activation of GPCRs through chemokines and prostaglandins leads to the activation of a variety of heterotrimeric G proteins [7]. Several studies have demonstrated the role of Gα_i_ family members in cell migration [8,9,10]. Previously, we showed that Gα_i_2 plays a critical role in oxytocin (OXT) and EGF signaling to induce cell migration in prostate cancer cells [11]. We also showed that Gα_i_2 acts at two distinct levels: first, its activation through specific GPCRs is required to induce cell migration and invasion in response to several stimuli, such as chemokines, transforming growth factor β1 (TGFβ1), and OXT [11,12]. These effects are, as expected, pertussis toxin-sensitive, and are upstream of the activation of the PI3-kinase signaling pathway [12]. Second, the knockdown and or knockout of endogenous Gα_i_2 in prostate cancer cells induced an attenuation of EGF-dependent cell migration and invasion [12]. These effects of Gα_i_2 in response to EGF are downstream of the activation of Phosphoinositide 3-Kinase (PI3K)/Akt/mammalian target of rapamycin (mTOR)/Rac1 cascade and are exerted at the levels of the formation of lamellipodia at the leading edge of migrating cells [12].

Lamellipodia formation is essential for cell migration in prostate cancer cells [13,14]. The activation of the WAVE2 and Arp2/3 complex, downstream of Rac1 activation, is required for actin polymerization, leading to the formation of lamellipodia, which initiate the directional migration of mammalian cells [15]. In the present study, we determined the effects of Gαi2 on cell migration downstream of Rac1 activation and its effects on the activation of Wave2 and Arp 2/3 in prostate cancer cells.

## 2. Results

### 2.1. Overexpression of Constitutively Active Gα_i_2 in PC3 Cells Increases Cell Motility

**Figure 1:**
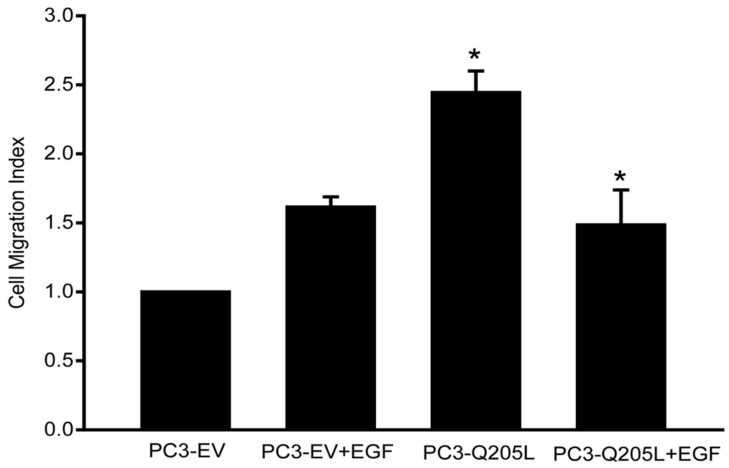
The overexpression of constitutively active Gα_i_2 in PC3 cells increases cell motility. PC3 cells were transfected with a pcDNA3.1 empty vector or constitutively active Gα_i_2 (Gα_i_2-Q205L). The upper panel shows a Western blot analysis of Giα2 in cell lysates to validate the knockdown of the protein. Migration assays were performed in the presence or absence of EGF (10 ng/mL). Results are expressed as a migration index, defined as the average number of cells per field for the ligand tested/the average number of cells per field for the vehicle control. Each bar represents mean ± SEM (*n* = 3). * Significantly different (*p* < 0.05) compared to PC3 cells overexpressing empty vector (PC3-EV).

To further investigate the role of Gα_i_2 protein on prostate cancer cell migration, we overexpressed a constitutively active form of Gα_i_2 (Gα_i_2-Q205L) in PC3 cells. As shown in Figure 1, the overexpression of Gα_i_2-Q205L significantly increased cell migration compared to cells transfected with control plasmids. These results suggest that the activation of Gα_i_2 in prostate cancer cells is sufficient to induce cell migration. Treatment with EGF also resulted in a significant increase in cell migration. However, the EGF effects on cell migration were of a lesser magnitude than those observed with the overexpression of Gα_i_2-Q205L and treatment with EGF significantly decreased the effects of Gα_i_2-Q205L (*p* < 0.05) overexpression when used in combination.

### 2.2. Gα_i_2 Inhibitor Blocked the Effects of Gα_i_2-Q205L on Cell Migration in PC3 Cells

**Figure 2:**
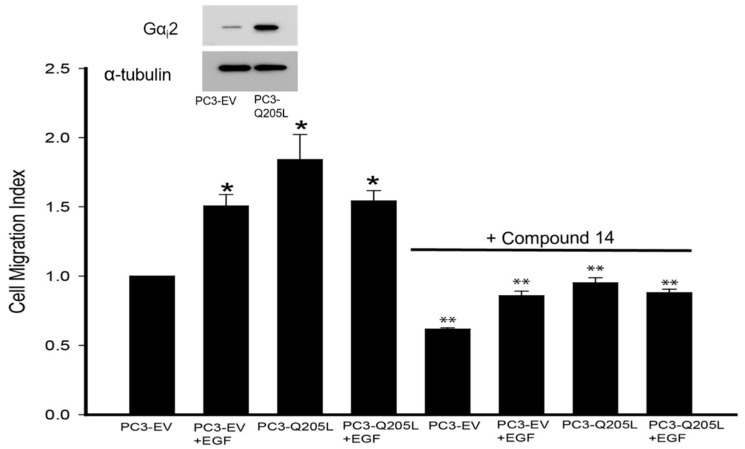
Gα_i_2 inhibitor blocked the effects of the overexpression of Gαi2-Q205L on cell migration in PC3 cells. Cell migration in control PC3-EV and PC3-Gα_i_2-Q205L cells was established after incubation with compound **14** at 10 μM, in the presence or absence of EGF (10 ng/mL). The upper panel shows a Western blot analysis of Gα_i_2 in cell lysates, to validate the overexpression of the protein. The results are expressed as a migration index. Each bar represents mean ± SEM (*n* = 3). * Significantly different (*p* < 0.05) compared to untreated PC3-EV cells. ** Significantly different (*p* < 0.05) when compared with the appropriate respective controls without the treatment with compound 14.

We have recently shown that small-molecule inhibitors targeting the Gα_i_2 protein are able to decrease cell migration in several cell lines, including prostate cancer cells [16]). To determine the effects of small-molecule inhibitors on our cell model, we transiently overexpressed constitutively active Gα_i_2 (Gα_i_2-Q205L) in PC3 cells and then performed migration assays using one of the most effective Gα_i_2 inhibitors, compound **14** [16]. As shown in Figure 2, the inhibitor blocked Gα_i_2-Q205L- and EGF-induced cell migration in PC3 cells.

### 2.3. Rac1 Inhibitor Does Not Block Migration in PC3 Cells Overexpressing Constitutively Active Gαi2

**Figure 3:**
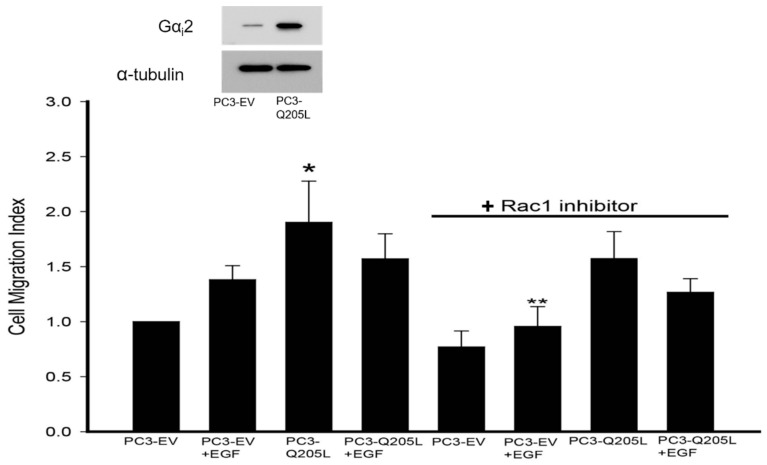
Rac1 inhibitor does not block migration in PC3 cells overexpressing constitutively active Gα_i_2. Cell migration in control PC3-EV and PC3-Gα_i_2-Q205L cells was studied after incubation with the Rac 1 inhibitor at 10 μM, in the presence or absence of EGF (10 ng/mL). The upper panel shows a Western blot analysis of Gα_i_2 in cell lysates to validate the overexpression of the protein. The results are expressed as a migration index. Each bar represents mean ± SEM (*n* = 3). * Significantly different (*p* < 0.05) compared to controls. ** Significantly different (*p* < 0.05) when compared with the same PC3 cell treatment groups without the Rac1 inhibitor.

In our previous studies, we showed that the overexpression of constitutively active Rac1 (Rac1Q61L) in PC3 cells increased cell migration in the cells compared to cell transfected with empty vectors (PC3-EV) [17]. When stimulated with EGF, there was no further increase in cell migration in these cells. Gα_i_2 knockdown resulted in the inhibition of cell migration in both control and Rac1Q61L-overexpressing PC3 cells [17]. In the current study, we overexpressed Gα_i_2-Q205L in PC3 cells, and we carried out cell migration assays in the presence of a specific Rac 1 inhibitor. As shown in Figure 3, the Rac1 inhibitor did not block increased cell migration in PC3 cells overexpressing constitutively active Gα_i_2. On the other hand, the Rac 1 inhibitor blocked the effects of EGF on cell migration.

### 2.4. PC3 Cells Overexpressing Constitutively Active Gαi2 Increase in Cell Size and Lamellipodia Formation

**Figure 4:**
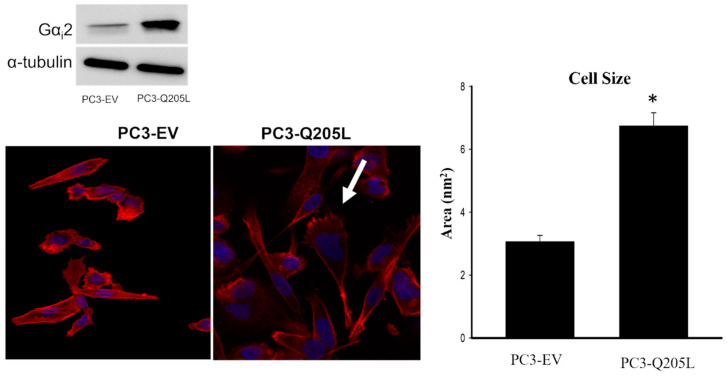
PC3 cells overexpressing constitutively active Gα_i_2 increase cell size and lamellipodia formation. PC3 cells were transfected with pcDNA3.1 empty vector and with the overexpression plasmid of the constitutively active form of Gα_i_2 protein (Gα_i_2-Q205L). Cells were stained for F-actin (red) and DAPI (blue). The arrow indicates lamellipodia at cell edges. Cells were visualized using a 20× objective scale = 20 μm. Right panel: Bar diagram of the quantitative analysis of cell area in PC3-overexpressing empty vector (PC3-EV) and PC3 cells overexpressing constitutively active Gαi2 (PC3-Q205L), determined using Zen Blue 3.1 version 1.1.2.0 Imaging software. * Significantly different (*p* < 0.05) compared to PC3-EV.

PC3 cells overexpressing Gα_i_2-Q205L were stained for F-actin and the nuclei using phalloidin/rhodamine and DAPI, respectively. As shown in Figure 4, PC3 cells overexpressing Gα_i_2-Q205L showed increased lamellipodia formation compared to the PC3-EV cells.

### 2.5. Rac1-Dependent Activation of Wave2 Is Impaired in the Absence of Gα_i_2

**Figure 5:**
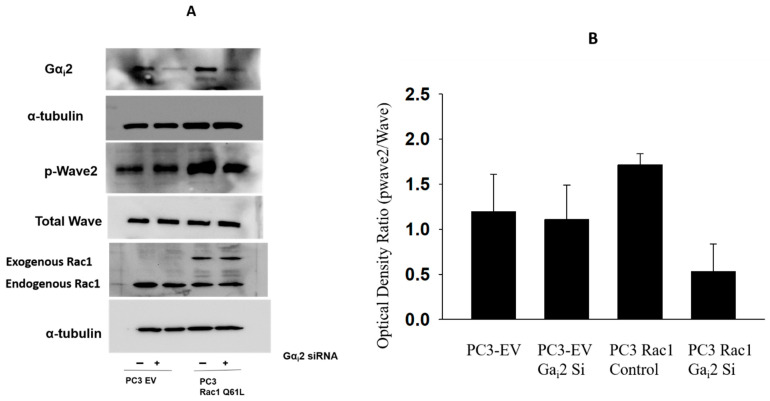
(**A**). Rac1-dependent activation of WAVE2 is impaired in the absence of Ga_i_2. Western blot analysis of Gα_i_2, p-Wave2 in PC3 cells constitutively overexpressing Rac1, with (+) or without (−) Gα_i_2 siRNA. Total WAVE2 and α-Tubulin were used as loading controls. (**B**). Ratios of optical densities (mean ± SEM) of Phosho-Wave2/total Wave2 after different treatments (data from three replicate experiments).

Previously, we showed that the overexpression of a constitutively active form of Rac1 (Rac1Q61L) in PC3 cells led to a significant increase in cell migration, which was not further increased in the presence of EGF compared to cells transfected with empty vectors (PC3-EV) [17]. Since the knockdown of endogenous Gα_i_2 by specific siRNAs resulted in the attenuation of basal and EGF-stimulated cell migration in both control and Rac1Q61L-overexpressing PC3 cells [17], we investigated the role of Gα_i_2 in the Rac1-dependent activation of Wave2 in PC3 cells overexpressing constitutively active Rac1 (PC3-Q61L) [17]. After transfection with scramble and Gα_i_2 siRNAs, total cell lysates were analyzed for phosphorylated and constitutive Wave 2 protein expression using Western blot analyses. As shown in Figure 5, the activation of the Rac1 downstream effector Wave2 was decreased after the knockdown of endogenous Gα_i_2 in PC3 cells overexpressing a constitutively active form of Rac1. These results suggest that the Gα_i_2 protein plays a crucial role during the activation of downstream proteins of Rac1.

## 3. Discussion

The novel findings in this manuscript show that activated Gα_i_2 plays a crucial role in the induction of cell migration in prostate cancer cells and this effect of Gαi2 does not require Rac1 activation. We also show that the Rac1-dependent activation of Wave2 protein is impaired in the absence of Gαi2.

We previously showed that EGF-induced cell migration is dependent on the activation of Gα_i_2 but does not depend on its activation through GPCR. These effects of Gαi2 are exerted at the level of lamellipodia formation and occur independently or downstream of the activation of PI3K/AKT/mTOR pathway. Furthermore, we showed that the overexpression of constitutively active Rac1 alone induced a significant increase in cell migration in PC3 cells and the knockdown of Gα_i_2 blocked induced cell migration in these cells [12]. Evidently, the Gα_i_2 protein is involved in cell migration at a step which is independent or downstream of Rac1 activation.

In the present study, the overexpression of constitutively active Gα_i_2 (Gα_i_2-Q205L) significantly increased cell migration in PC3 cells and the use of a specific Rac1 inhibitor did not block increased cell migration in these cells. We also observed differences in the effects of the overexpression of constitutively active Gα_i_2 and treatment with EGF. EGF alone was less potent in inducing cell migration and it also inhibited the effects of constitutively active Gα_i_2 when used in combination. Furthermore, the effects of EGF on cell migration were blocked by the Rac1 inhibitor, indicating that EGF effects are exerted upstream of Rac1 activation. On the other hand, our results suggest that constitutively active Gα_i_2 increases cell migration in PC3 cells in the absence of Rac1 activation, suggesting a role of Gα_i_2, independently or downstream of Rac1, in cell migration.

At the initiation of cell migration, actin assembles at the leading edge, determining the direction of the cell movement [18]. At the migration front, the activation of cell surface receptors, such as integrins, leads to the activation of the Rho-family GTPases, creating a cascade effect by activating the phosphatidylinositol biphosphate pathway, the WASP/Scar proteins, and the Arp2/3 complex, which guide the formation of new actin filaments branching from the preexisting ones [19,20,21,22,23]. This process leads to the cell membrane pushing forward, leading to the formation of lamellipodia The PI3K signaling pathway is involved in lamellipodia formation, and it was shown that the GPCR-dependent activation of Gα_i_2 is required for the activation of PI3 kinase pathway in response to TGFβ1 and OXT [12]. Whether PI3K activation is also required downstream of Gαi2 to induce the formation of lamellipodia and induction of cell migration after the overexpression of constitutively active Gα_i_2 remains to be determined.

Rac1 is a GTP-binding protein with low molecular weight, belonging to the Rac subfamily of the Rho GTPase family [24,25,26]. As a key molecule in regulating cell migration, Rac1 participates in signal transduction from the extracellular stimuli to the actin cytoskeleton and promotes the establishment of cell polarity, which plays a key role in lamellipodia formation and cell migration [24,25,27,28]. Previous studies show that the absence of Gα_i_2 resulted in a decrease in lamellipodia formation and a decrease in cell size in PC3 cells overexpressing constitutively active Rac1 [12]. In the current study, the overexpression of constitutively active Gα_i_2 in PC3 cells leads to a prevalent formation of lamellipodia and a directed cell migration when compared to control PC3 cells.

The Scar/Wave complex, consisting of five subunits, is a key factor promoting the formation of actin-rich protrusions in migrating cells. As an actin-related protein, the 2/3 (Arp2/3) activator stimulates actin polymerization through the Arp2/3 complex to generate lamellipodia and pseudopodia [29,30,31,32]. After receiving upstream signals from Rac1, the Scar/Wave complex regulates F-actin cytoskeleton polymerization by activating the Arp2/3 complex, thus forming actin-based membrane processes, which are essential for cell migration and cancer cell invasion [24,25,33,34]. In an attempt to understand the involvement of Gα_i_2 in the activation of the downstream effectors of Rac1, we knocked down Gα_i_2 in PC3 overexpressing constitutively active Rac1. The Rac1-dependent activation of Wave2 became impaired in the absence of the Gα_i_2 protein. We concluded that Gα_i_2 plays a role in activating the proteins downstream of Rac1 to induce lamellipodia formation at the leading edge of migrating cells and cell migration. Based on the results of the current study, we conclude that Gα_i_2 plays a crucial role in lamellipodia formation and cell migration in prostate cancer cells, and these effects do not depend on the activation of the Rac1 protein. Further studies are needed to identify the cellular and molecular mechanisms that are GPCR-independent and the essential effects of Gαi2 at the level of lamellipodia formation and the induction of cell migration in prostate cancer cells. The inhibition of these mechanisms may provide tools to inhibit cell motility, invasion, and metastasis in prostate cancer cells.

## 4. Materials and Methods

### 4.1. Cell Culture Chemicals and Reagents

PC3 prostate cancer cells were obtained from American Type Culture Collection (ATCC) (Rockville, MD USA). PC3 cells are an androgen-independent cell line derived from bone metastasis. They were maintained in a 5% CO_2_ environment at 37 °C, as previously described [12]. Anti-phospho-WAVE2 (Ser343) (Cat#07-1512) was obtained from EMD Millipore (Temecula, CA, USA). WAVE2 (Cat# WP1791) were obtained from ECM Biosciences (Versailles, KY, USA). Purified Mouse Anti-Rac1 antibody (Cat# 610650) was obtained from BD Transduction Laboratories (New Jersey, NY, USA). Mouse anti-α-tubulin and bovine serum albumin (BSA) were obtained from Sigma Chemicals (St. Louis, MO, USA). Rat tail collagen, and transwell inserts were obtained from BD Biosciences (San Jose, CA, USA). Control (sc-37007) and Gα_i_2-targeting (sc-41752) siRNAs, and transfection reagent (sc-29528), were purchased from Santa Cruz Biotechnology (Dallas, TX, USA). Alexa Fluor^®^ 488 goat anti-rabbit IgG (H+L) antibody and epidermal growth factor (EGF) were obtained from Life Technologies (Grand Island, NY, USA). Rhodamine phalloidin was purchased from Cytoskeleton, Inc. (Denver, CO, USA). Lipofectamine 3000 Transfection Reagent and 4′,6-diamidino-2-phenylindole (DAPI) were obtained from Thermo Fisher Scientific Inc. (Waltham, MA, USA). Cell culture reagents were purchased from Corning Life Sciences (Tewksbury, VA, USA).

### 4.2. Transient Transfection with Gα_i_2 siRNA

PC3 cells were seeded in 6-well plates at a density of 1.5 × 10^5^ cells per well and transfected with control or Gα_i_2-specific siRNAs, as previously described [11,12], according to the manufacture’s protocol. Briefly, media with no antibiotics (200 µL/well) containing 30 nmol/L of siRNA were mixed with the transfection reagent (6 µL/well) and, after incubation, the siRNA mixtures were added drop-by-drop to the cells. The transfection media were replaced with the regular culture media (2 mL/well) the next day. Cells were harvested 48 h after transfection and used for different assays.

### 4.3. Overexpression of Constitutively Active Gα_i_2 (Gα_i_2-Q205L)

PC3 cells were seeded in 10 cm^2^ culture plates at a density of 1.0 × 10^6^ cells per plate. The next day, cells were transfected with pcDNA3.1 empty vector or with the overexpression plasmid of constitutively active Gα_i_2 gene (pcDNA3.1-Gα_i_2-Q205L; cDNA Resource Center, Bloomsberg, PA, USA), using the Lipofectamine 3000 Transfection reagent, following the manufacturer’s protocol. Briefly, 1 μg of plasmid DNA was diluted with MEM and incubated for 15 min in the presence of the transfection reagent. After the incubation time, the complex DNA/transfection reagent (ratio 1:3) was added to the cells. After 72 h, the cells were harvested, and migration assays and Western blots were performed.

### 4.4. Western Blot Analysis

Western blot analyses were performed as described previously [12]. Briefly, protein samples (30–35 µg proteins) were separated on 10% SDS-PAGE gels and transferred to polyvinylidene difluoride membranes (Millipore Corp., Bedford, MA, USA). After blocking, the membranes were incubated with appropriate dilutions of specific primary antibodies (1:1000 for Wave2, Rac1; 1:5000 for Gα_i_2; 1:500 for p-Wave2; 1:3000 for α-tubulin) overnight at 4 °C. After washing, the blots were incubated with appropriate secondary antibodies and developed in ECL mixture. The blots were visualized using BioRad ChemiDoc Imaging System, according to the manufacturer’s instructions. The density of specific protein bands was determined using ImageJ (version 1.45) software (NIH, Bethesda, MD, USA) and normalized using density of α-tubulin bands, which was used as a loading control.

### 4.5. Cell Migration

In vitro cell migration assays were conducted using 24-well transwell inserts (8 μm), as described previously [12]. Briefly, transwell inserts were coated with rat tail collagen (50 mg/mL). A total of 1.5 × 10^5^ cells/well were seeded inside the transwell inserts, and EGF (10 ng/mL) was used as chemoattractant solution. The plates were incubated at 37 °C for 5 h. After fixation, the cells were stained with 3 ng/mL of DAPI, images of five non-overlapping fields were captured using Axiovert 200M, Carl Zeiss (Göttingen, Germany) microscope, and the number of stained nuclei was determined via automatic counting using image analysis software (ZEN 2012; Carl Zeiss Microscopy, LLC, White Plains, NY, USA). The results were expressed as a migration index, defined as follows: the average number of cells per field for test substance/the average number of cells per field for the medium control.

### 4.6. Immunofluorescence and Actin Staining

PC3 cells with or without overexpression of constitutively active Gα_i_2 (Gα_i_2-Q205L) were grown (1.5 × 10^5^ cells/mL) on glass coverslips for 48 h, fixed with 4% paraformaldehyde in phosphate-buffered saline (PBS) for 15 min and washed with PBS three times. Cells were permeabilized with 0.1% Triton X-100 in PBS for 10 min and incubated with 10% normal goat serum for 1 h to block nonspecific antibody binding. Then, the cells were incubated with anti-G_i_α2 antibody (1:500) overnight at 4 °C. After washing, the cells were incubated with a secondary antibody, Alexa Fluor 488-conjugated anti-rabbit immunoglobulins (1:1000), for 45 min. To validate the specificity of the antibodies, parallel cell preparations were incubated with either primary or secondary antibodies alone and processed as negative controls. The cells were washed with PBS and incubated with Rhodamine–phalloidin for 30 min to detect F-actin filaments and DAPI for 10 min to detect the nuclei, and mounted on Vectashield mounting medium (Vector Laboratories, Burlingame, CA, USA). Images were captured using Zeiss LSM 700 Confocal Microscope (Carl Zeiss Microscopy LLC, White Plains, NY, USA) with a 20× magnification objective. The cell size was determined using ZEN Blue 3.1 (version 1.1.2.0) imaging software (Carl Zeiss Microscopy LLC, White Plains, NY, USA). The cell size was determined in three random fields (5–10 cells) in PC3 cells overexpressing the pcDNA3.1 empty vector or overexpression plasmid of the constitutively active form of Gα_i_2 protein (Gα_i_2-Q205L). The differences in cell size between the two groups were analyzed using Student’s “t” test. The cell area is presented as nm^2^.

### 4.7. Statistical Analysis

All experiments were repeated at least three times using different cell preparations. The results are presented as the mean ± SEM of three replicate experiments and images from a single representative experiment are presented. ANOVA and Duncan’s modified multiple range tests were employed to assess the significance of differences among various treatment groups (*p* < 0.05).

## 5. Conclusions

On the basis of the results presented in this manuscript, we conclude that the essential role of Gα_i_2 in cell migration and invasion in response to multiple extracellular stimuli is exerted at a level downstream of Rac 1. Furthermore, Gα_i_2-induced cell migration does not require Rac 1 activation.

## Data Availability

The data presented in this article are available.
